# Astragaloside IV improves the pharmacokinetics of febuxostat in rats with hyperuricemic nephropathy by regulating urea metabolism in gut microbiota

**DOI:** 10.3389/fphar.2022.1031509

**Published:** 2022-12-20

**Authors:** Zhen Xiong Zhao, Xiao Hui Tang, Sheng Lu Jiang, Jia Qian Pang, Yu Bin Xu, Dan Dan Yuan, Ling Ling Zhang, Hui Min Liu, Qing Fan

**Affiliations:** ^1^ Taizhou Central Hospital (Taizhou University Hospital), Taizhou, Zhejiang, China; ^2^ Shandong Cancer Hospital and Institute, Shandong First Medical University and Shandong Academy of Medical Sciences, Jinan, Shandong, China

**Keywords:** hyperuricemic nephropathy, gut microbiota, febuxostat, astragaloside IV, uric acid

## Abstract

Hyperuricemic nephropathy (HN) is a common clinical complication of hyperuricemia. The pathogenesis of HN is directly related to urea metabolism in the gut microbiota. Febuxostat, a potent xanthine oxidase inhibitor, is the first-line drug used for the treatment of hyperuricemia. However, there have been few studies on the pharmacokinetics of febuxostat in HN animal models or in patients. In this study, a high-purine diet-induced HN rat model was established. The pharmacokinetics of febuxostat in HN rats was evaluated using LC-MS/MS. Astragaloside IV (AST) was used to correct the abnormal pharmacokinetics of febuxostat. Gut microbiota diversity analysis was used to evaluate the effect of AST on gut microbiota. The results showed that the delayed elimination of febuxostat caused drug accumulation after multiple administrations. Oral but not i. p. AST improved the pharmacokinetics of febuxostat in HN rats. The mechanistic study showed that AST could regulate urea metabolism in faeces and attenuate urea-ammonia liver-intestine circulation. Urease-related genera, including *Eubacterium*, *Parabacteroides*, *Ruminococcus*, and *Clostridia*, decreased after AST prevention. In addition, the decrease in pathogenic genera and increase in short-chain fatty acids (SCFA) producing genera also contribute to renal function recovery. In summary, AST improved the pharmacokinetics of febuxostat in HN rats by comprehensive regulation of the gut microbiota, including urea metabolism, anti-calcification, and short-chain fatty acid generation. These results imply that febuxostat might accumulate in HN patients, and AST could reverse the accumulation through gut microbiota regulation.

## Introduction

Hyperuricemic nephropathy (HN) is a common clinical complication of hyperuricemia (Dalbeth et al., 2021; Xiong et al., 2021). Excessive urinary acid deposition in the kidneys traditionally induces hyperuricemic nephropathy (Johnson et al., 2018). Studies have shown that hyperuricemia leads to kidney injury *via* angiotensin system activation, oxidative stress, tubular epithelial cell transition, and inflammation (Braga et al., 2020; Ejaz et al., 2020; Wen et al., 2021). Interestingly, studies have shown that the pathogenesis of HN is closely related to the gut microbiota (Han et al., 2020; Pan et al., 2020; García-Carrasco et al., 2021). Uric acid, mainly produced in the liver, is the end product of dietary and endogenous purine metabolism. Approximately 600–700 mg of uric acid are produced and excreted daily by the human body. Nearly 2/3 is excreted through the kidney and 1/3 *via* the intestine (Dalbeth et al., 2021). Once the kidney is damaged, the intestine mainly eliminates urinary acid as compensation. Simultaneously, hyperuricemia may cause an imbalance in the nitrogen metabolism of the gut microbiota (Pan et al., 2020). Therefore, investigating the relationship between intestinal bacteria and the pathogenesis of HN may help identify potential therapeutic targets.

The human gut microbiota consists of over 1,900 species of bacteria, more than 10 times the number of cells in the human body. In addition to metabolism, the gut microbiota also plays a role in maintaining homeostasis of the intestinal environment and can be explored as a potential target for the personalized medication (Zhao et al., 2018). Many studies have focused on the interactions between gut microbiota and drug intervention. The gut microbiota can produce small molecules that have been found to be an important part of uremic toxins, including ammonia, urea, and amines (Wikoff et al., 2009). Moreover, gut microbiota changes in composition and function are closely related to the progression of chronic kidney disease because of purine and uric acid metabolism by gut microbiota (Pan et al., 2020). Therefore, the gut microbiota may be a potential target for treating HN.

Febuxostat, a potent xanthine oxidase inhibitor, is the first-line drug for hyperuricemia therapy (White et al., 2018). However, there are few reports on the pharmacokinetic characteristics of febuxostat in HN animal models or in patients. In addition, renal damage may affect the clearance rate of drugs (Fang et al., 2021). It was reported that febuxostat was used to treat gout and chronic kidney disease (CKD), and no accumulation was found in slight renal injury patients (Stamp et al., 2021). However, it has not been reported whether febuxostat accumulates in patients with severe renal injury. Therefore, we investigated the pharmacokinetics of febuxostat in HN rats to facilitate the rational use of febuxostat.

Astragaloside IV (AST) is a natural product and a major active component of *Astragalus* membranaceus (Wei et al., 2020). AST reduces renal injury by inhibiting ferroptosis (Qin et al., 2022), oxidative stress (Du et al., 2018), and transforming growth factor-β (TGF-β) (Gao et al., 2020; Wang et al., 2020). In addition, renal fibrosis and amelioration of HK-2 cell apoptosis were inhibited by AST (Cao et al., 2019; Wang and Guo, 2019). However, AST, a natural glycoside compound, has low oral bioavailability (<2.5%) (Gu et al., 2004), making it difficult to maintain an effective concentration in the target organ. Furthermore, unabsorbed drugs interact with the gut microbiota in the intestine and may induce changes in the intestinal environment, altering the gut microbiota (Wang et al., 2021). Overall, it is important to explore the therapeutic effect of AST on HN by regulating the gut microbiota.

Hence, in this study, an HN rat model was established and used to investigate the therapeutic effect of AST *via* regulating the gut microbiota. The pharmacokinetic profile of febuxostat as the probe to evaluate renal injury in HN was determined using LC-MS/MS. The gut microbiota was analyzed using 16 S ribosomal RNA (16 S rRNA) to explore the effect of AST on the regulation of gut microbiota.

## Materials and methods

### Chemicals and reagents

Astragaloside IV (>98%) was obtained from Yirui Biotechnology Co., Ltd. (Chengdu, China). Febuxostat (99%) was bought from TLC Pharmaceutical Standards Ltd. (Ontario, Canada). Febuxostat-d9 (>97%) obtained from Toronto Research Chemicals (Toronto, Canada) was used as an internal standard (IS) in LC-MS/MS analysis. High performance liquid chromatography (HPLC) grade acetonitrile and methanol were purchased from Thermo Fisher Scientific Co., Ltd. (Fair Lawn, United States). Ultra-pure water was obtained from Hangzhou Wahaha Group Co., Ltd. (Hangzhou, China). Adenine, yeast powder urease activity test kit, hematoxylin-eosin staining (H&E) staining kit, Masson staining kit, urea and uric acid standard were purchased from Beijing Solarbio Science and Technology Co., Ltd. (Beijing, China). The urea nitrogen, blood ammonia and creatinine test kits were purchased from Nanjing Jiancheng Bioengineering Institute (Nanjing, China).

### Animals

Male Sprague-Dawley (SD) rats (150 ± 5 g) were obtained from Zhejiang Vital River Company (Hangzhou, China). They were kept under 12 h light/dark cycles and allowed free access to food and water. The experiment was carried out in strict accordance with the ethical guidelines for experimental animals, and was approved by the Animal Ethics Committee of the Experimental Animal Institute of Taizhou University.

### Group information and sample collection

Thirty six rats were randomly divided into 6 groups, and the group information was as follows: Group 1, normal group (fed with normal food); Group 2, model control group with slightly renal injured (fed with a special folder containing 10% yeast and 0.15% adenine for 4 weeks); Group 3, model control group with severe renal injured (fed with a special folder containing 10% yeast and 0.15% adenine for 8 weeks; Group 4, AST 5 mg/kg treatment group (AST-L), which was treated with AST 5 mg/kg/d p. o. for 8 weeks; Group 5, AST 10 mg/kg treatment group (AST-H), which was treated with AST 10 mg/kg/d p. o. for 8 weeks; Group 6, AST i. p. Treatment group (AST-IP), which was treated with AST 2 mg/kg/d i. p. for 8 weeks; Group 4–6 were fed with a special folder containing 10% yeast and 0.15% adenine for 8 weeks during the treatment. Serum of rats from group 1 and 2 were collected after 4-week treatment. Serum and faeces from group 3–6 were collected after 8-week treatment. After the rats were anesthetized, the kidneys were taken out and were fixed in 4% tissue cell fixative (4% paraformaldehyde) for 24 h, dehydrated by automatic dehydrator for 16 h, and then routinely embedded in a paraffin embedding machine for preparation of kidney tissue sections. Kidney tissue sections were then subjected to H&E staining and Masson staining.

### H&E and masson staining

The pre-prepared paraffin sections were dewaxed for 5 min with xylene I and II, respectively, and then treated with ethanol gradient treatment (anhydrous ethanol for 5 min, 95% ethanol for 2 min, 80% ethanol for 2 min, 70% ethanol for 2 min). After 2 min washed by distilled water, the dewaxed tissue sections were stained with hematoxylin dye for 20 min, and then rinsed with tap water. Next, the differentiation solution was applied for 30 s, and the tissue samples were soaked in water for 15 min. Then dye the sample with eosin staining solution for 30 s and rinse in tap water. After soaking in water for 5 min, the sample was dehydrated by ethanol gradient, then xylene was transparent and sealed with neutral glue. The samples were observed by an optical microscope.

Masson staining procedure was as following: the paraffin sections were dewaxed using xylene. After dyed by Weigert iron hematoxylin staining solution for 5 min, the acidic ethanol differentiation solution was used for differentiation for 5 s, and washed with water. Masson blue solution was applied to return to blue for 3 min and then the samples were rinsed with distilled water for 1 min. After this, Lichunhong Magenta staining solution were used to stain the samples for 5 min, and then a weak acid working solution (mixed solution of distilled water and weak acid solution with a ratio of 2:1) was used to wash the samples for 1 min, next, phosphomolybdic acid solution for 2 min and weak acid working solution again for 1 min. Samples were then stained by aniline blue solution for 2 min, and washed with weak acid working solution for 1 min. Then samples were dehydrated with 95% ethanol rapidly and then absolute ethanol for 3 times. After xylene transparency for 3 times, the samples were sealed with neutral gum. Finally, an optical microscope was used to observe and photograph.

### Creatinine, urea nitrogen, and ammonia measurement in serum

The serum creatinine, urea nitrogen (BUN) and ammonia levels were determined to evaluate the renal function. Blood samples (200 μl) from rats were centrifuged at 2,000 rpm for 5 min, and the serum samples were isolated to be tested using detection kit.

### Pharmacokinetic study

Pharmacokinetic studies of febuxostat (10 mg/kg, oral) in SD rats were carried out in different groups. The pharmacokinetic experiment was performed in group 1 and 2, 1 month after modeling. Group 3–6 were undergone the above operations after the 2 month treatment. The febuxostat was dissolved in a 0.9% NaCl-injectable solution and Tween 80 (9:1, v/v) at the concentration of 2 mg/ml, and was administered orally to the rats. The blood samples were collected from fundus vein at 0, 0.25, 0.5, 1, 1.5, 2, 3, 4, 8, 12, 24, and 48 h post-dosing and the plasma samples were centrifuged at 3000 rpm for 10 min and stored frozen at −80°C until LC-MS/MS analysis. After single dose administration, the rats were treated with febuxostat (10 mg/kg/day, oral) for continual 7 days. The blood samples were collected from fundus vein at 0, 0.25, 0.5, 1, 1.5, 2, 3, 4, 8, 12, 24, and 48 h post-dosing on the last day of administration, and the plasma samples were centrifuged at 3000 rpm for 10 min and stored frozen at −80°C until LC-MS/MS analysis.

### Sample preparation

A stock solution of febuxostat was prepared by dissolving 10 mg l-malic acid in 10 ml methanol as solution to be assayed, with the concentration of 1 mg/ml. Appropriate dilutions were made in methanol for febuxostat to produce working stock solution of 200, 100, 50, 10, 5, 1 and 0.5 μg/ml. The stock solution of febuxostat-d9 was prepared using methanol at the concentration of 10 μg/ml, using as IS. The QC stock solutions of febuxostat were set at 160 (HQC), 10 (MQC), 1 (LQC) and 0.5 (LLOQ) μg/mL. The plasma samples were taken 50 μl out into a tube, and then 5 μl of IS stock solution was added into the tube. Methanol (200 μl) was added into the tube as protein precipitant. After 30 s vortex mixing, the sample was centrifuged at 10,000 rpm for 10 min. The supernatant was taken out for LC-MS/MS detecetion. Calibration standards and QC samples were prepared by spiking 50 μl of control pooled rat plasma with the appropriate working solution of febuxostat (5 μl). The calibration curve was developed by plotting the ratio to the peak area of febuxostat to that of IS *versus* the nominal concentration of calibration standards.

### Instruments

The sample detection was accomplished using a Shimadzu LC-MS/MS 8050 system (Tokoy, Japan). The chromatographic separation was carried out using an Eclipse XDB-C18 column (150 × 4.6 mm, 3.5 μm). The mobile phases were 5 mM ammonium formate with 0.2% formic acid in water (Phase A) and acetonitrile (Phase B). Gradient elution method was adopted with the mobile phase ratio: 0 min, A: B = 10 : 90; 1 min, A: B = 10 : 90; 3 min, A: B = 80 : 20; 3.01 min, A: B = 10 : 90, and the total analysis time was 5 min. The flow rate was set at 0.5 ml/min, and the column temperature was 30°C. The mass spectrometer was operated in positive ion and multiple reaction monitoring (MRM) acquisition modes. An ESI resource was used with the interface temperature of 250°C, DL temperature of 300°C and heat block temperature of 400°C. The quantitative ion pairs were 315.30→271.25 m/z for febuxostat and 324.30→280.30 m/z for febuxostat-d9.

### Method validation

The bioanalytical method was validated for febuxostat in rat plasma as per the FDA guideline. The parameters evaluated were linearity, precision, accuracy, recovery, and matrix effectcs.

### Microbial diversity analysis

At the end of the 2 months model establishment and AST treatment, the fecal samples of rats within 24 h were collected in a metabolic cage, which can complete separate the faeces and urine. Then the samples were freezed under −80 °C. The V3-V4 region of the bacteria’s 16 S rRNA gene was amplified with primers 338F and 806R. AxyPrep DNA GelExtraction Kit (Axygen Biosciences, UnionCity, CA, United States) was used to purify the amplicons, followed by quantification using QuantiFluor-ST (Promega, Madison, WI, United States). Illumina MiSeq instrument (Illumina, San Diego, CA, United States) was used for sequencing. The 16 S rRNA sequencing data were analyzed with Quantitative Insights Into Microbial Ecology platform (V.1.9.1). Operational taxonomic units (OTUs) with similarity over 97% were selected for taxonomy identification with Greengenes database (V.13.8).

### Urease, urea and uric acid detection in faeces

Fecal samples (0.2 g) were mixed by 1 ml PBS buffer, and they were filtered by sterile gauze. The filtrate was centrifuged at 5000 rpm for 15 min at −4°C, and then the supernatant was remove. The intestinal bacteria in sediment was washed twice by cold PBS buffer and the final volume was set at 0.2 ml. The urease in faeces was measured by the urease kit from Solarbio. The detection of urea and uric acid was operated using LC-MS/MS. Chromatographic separation was carried out using an Alltima C18 (150 mm × 4.6 mm, 5 mm) column. The linear gradient elution flow rate was 0.4 ml/min, with water as mobile phase A, and methanol as mobile phase B: 1 min (95% A and 5% B), 2 min (80% A and 20% B), 4 min (5% A and 95% B), 7.00 min (5% A and 95% B), 7.01 min (5% A and 95% B), 13 min stop. The mass spectrometer was operated in a multiple reaction monitoring (MRM) mode. The following product ion precursors were monitored: urea (m/z) 61.10 [M + H]^+^→44.10, IS for urea (hydroxyurea) (m/z) 77.20 [M + H]^+^→44.10; uric acid (m/z) 167.25 [M-H]^-^ →124.15, IS for uric acid (6-Benzylaminopurine) (m/z) 226.20 [M-H]^-^→65.10. The ESI temperature was consistent with the method adopted in febuxostat detecetion.

### Statistical analysis

The pharmacokinetic parameters were calculated by a non-compartmental method using DAS Version 2.0 (Bioguider, Shanghai, China). The statistical analyses were conducted using two-way ANOVA and Student’s t test with GraphPad Prism Version 8.01 (GraphPad Software, CA, United States). The data are expressed as the means ± standard deviation. *p* values < 0.05 were considered statistically significant.

## Results

### Hyperuricemia nephropathy model establishment

**FIGURE 1:**
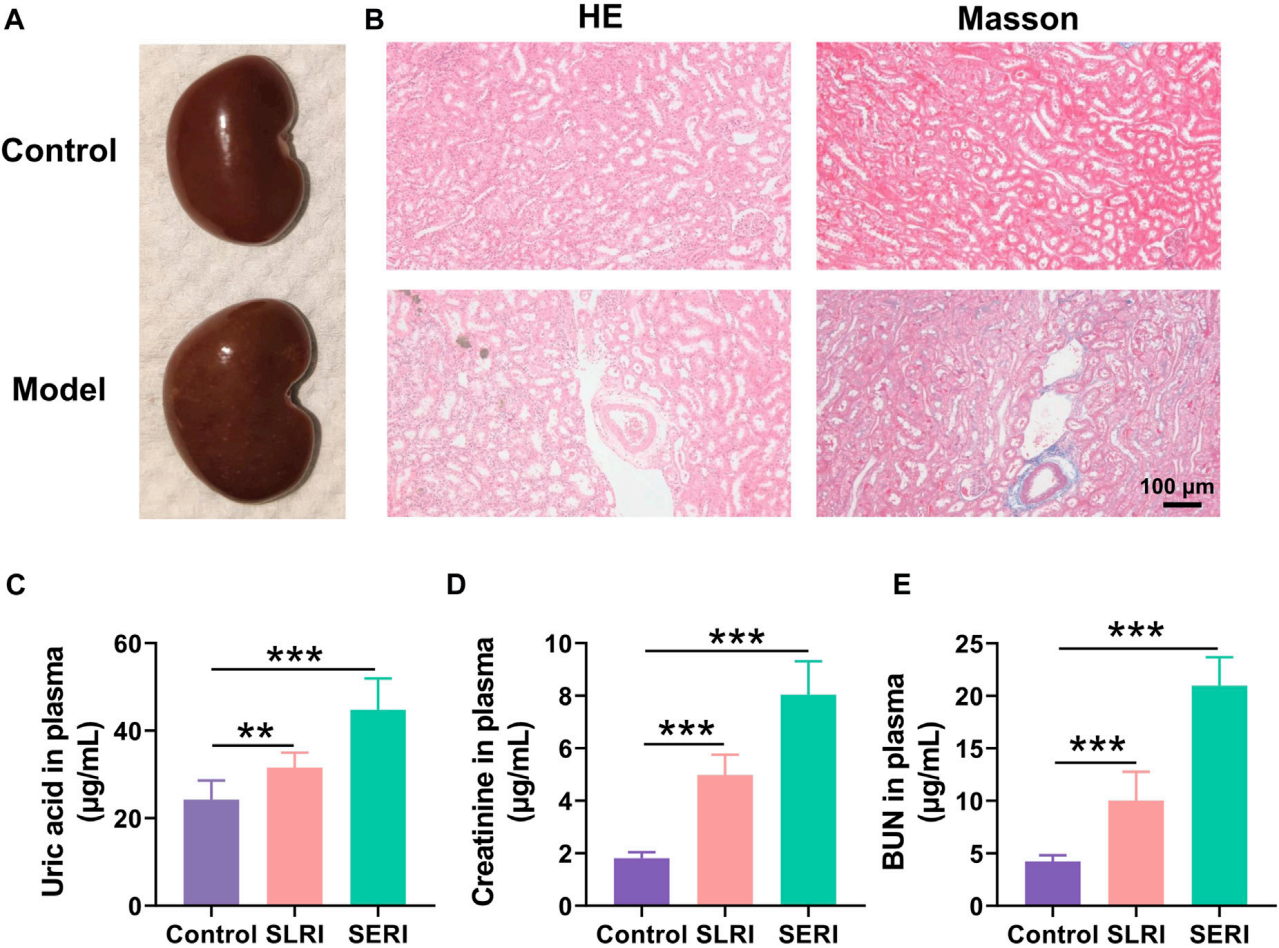
Biological index for model establishment. **(A)** Normal kidney morphology; **(B)** H&E pathological staining and Masson staining; **(C)** The level of uric acid in normal group and model group; **(D)** the level of creatinine content in normal group and model group; **(E)** urea nitrogen (BUN) content in normal group and model group. Data are expressed as mean ± SD (*n* = 6), **p < 0.01, ***p < 0.001 vs. CON group.

**FIGURE 2:**
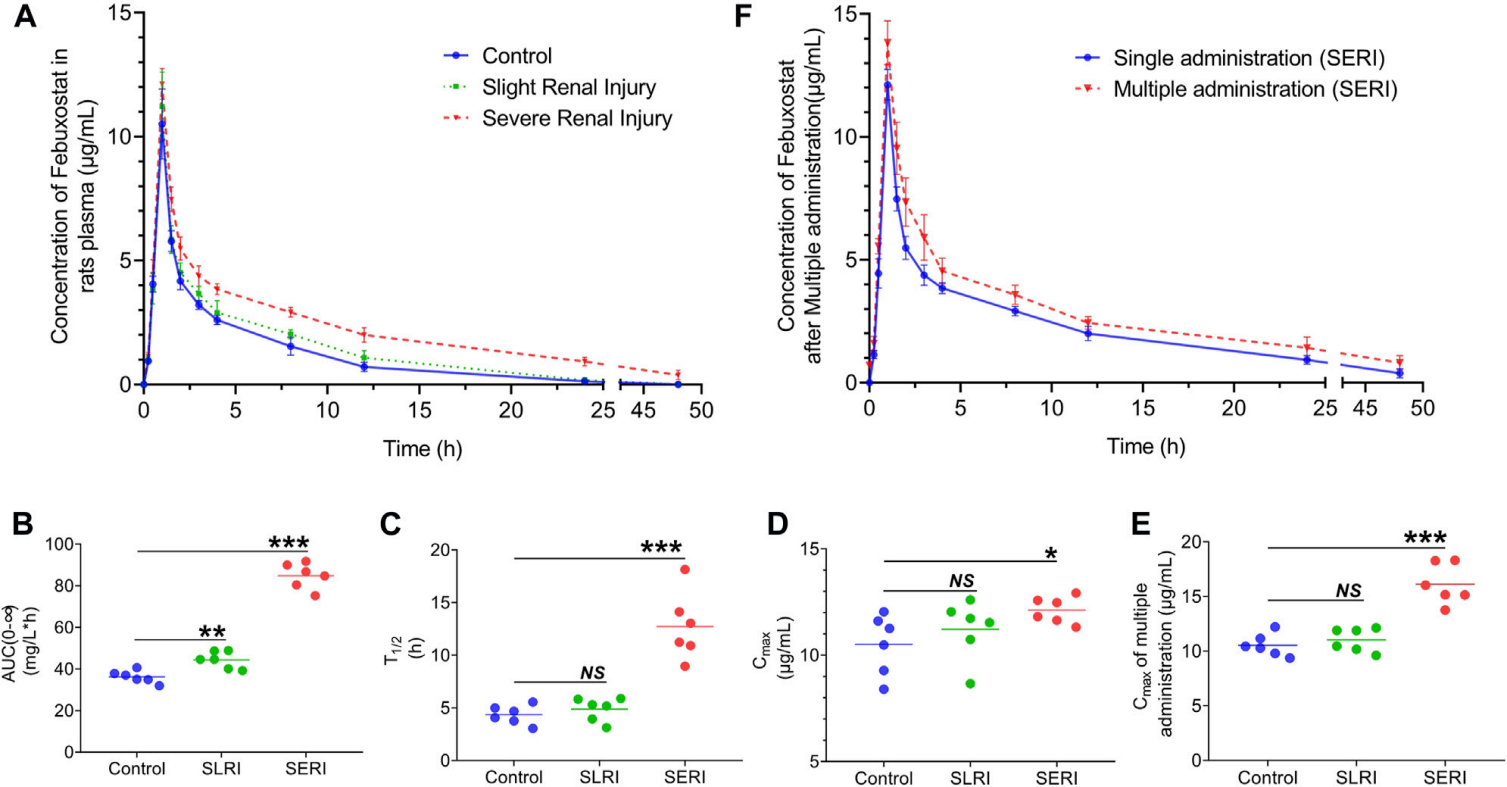
Pharmacokinetics of febuxostat in normal and HN rats. **(A)** Drug concentration-time curve of febuxostat in normal group, SLRI group and SERI group; **(B)** the value of AUC_(0-∞)_ of three groups; **(C)** the value of T_1/2_ of three groups; **(D)** the value of C_max_ of three groups; **(E)** the value of C_max_ of three groups after multiple administration; **(F)** Pharmacokinetics of febuxostat in SERI rats after multiple administration. NS represented no significant differences, *p < 0.05, ***p < 0.001 vs. control group.

The model of hyperuricemia nephropathy induced by a high-purine diet was established by administering a diet containing 10% yeast powder and 0.15% adenine for 4–8 weeks. The serum uric acid increased significantly, in a time-dependent manner, during the high-purine diet (Figure 1C), impairing renal function. Creatinine and BUN levels in the serum also increased significantly (Figures 2D,E). The pathological results indicated the same situation. The kidneys of the normal group were light brown with a smooth surface. In contrast, the kidneys of the model group became dim, increased in volume, and showed white spots on the surface (Figure 1A). H&E staining results showed that the normal rats had a normal renal structure and no uric acid crystallization (Figure 1B). In contrast, the uric acid crystals in the lumen and interstitium of the renal cortex were observed as a solid structure in the model group kidneys. Moreover, the renal tubules were vacuolar-degenerated, and the interstitium was significantly broadened. Masson staining results showed that the renal interstitium was broadened, and fibrosis was observed in SD rats.

### Abnormal pharmacokinetics of febuxostat in HN rats

The pharmacokinetics of febuxostat was evaluated in both normal and HN rats. Febuxostat (10 mg/kg) was orally administered to normal rats, slightly renal-injured rats (SLRI, high-purine diet for 4 weeks), and severe renal injury rats (SERI, high-purine diet for 8 weeks). The analysis of biological samples was conducted using an LC-MS/MS method. The method validation results are shown in the supplementary material (Supplementary Tables S1, S2), indicating that the method was sensitive and accurate. The pharmacokinetic results are shown in Figure 2A. The drug concentration-time curves for the three groups showed similar tendencies, with T_max_ of 1 h. Significantly, febuxostat concentration in HN rats increased to different levels in the elimination phase. Febuxostat in SERI rats did not completely eliminate at 48 h, indicating that HN might lead to impaired metabolism of febuxostat. The pharmacokinetic parameters are shown in the supplementary material (Supplementary Table S5). The AUC, T_1/2_, and C_max_ changed significantly in both the SERI and SLRI groups (Figures 2B–D). The AUC of SLRI rats was significantly higher than that of normal rats, while T_1/2_ and C_max_ showed no significant difference compared to those of the control group, indicating that SLRI rats were in the early stage of renal injury. SERI rats with significantly higher AUC, T_1/2_, and C_max_ than normal rats suffered from severe renal injury. The results of febuxostat pharmacokinetic in SERI rats after multiple administration were shown in Figures 2E,F. The pharmacokinetic parameters were shown in supplementary material (Supplementary Table S6). The AUC and C_max_ of the multiple administration were significantly higher than those of single administration, which indicated that febuxostat in SERI rats accumulated after 7 days multiple administration.

### Astragaloside IV attenuates hyperuricemia-induced functional and structural renal damage

**FIGURE 3:**
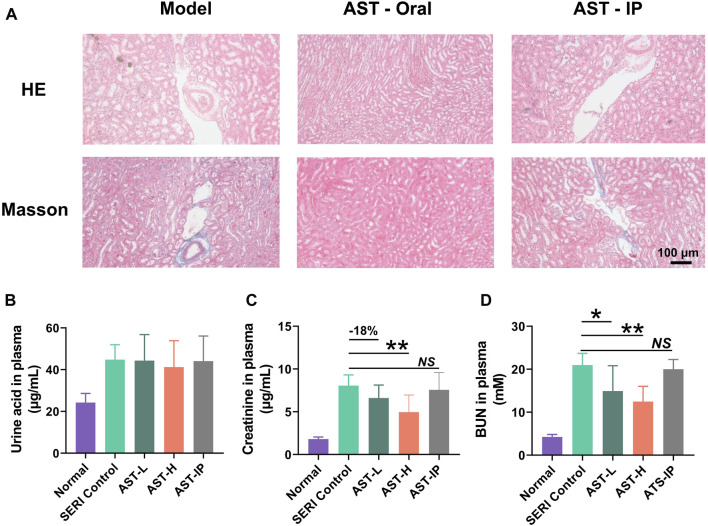
AST reduced hyperuricemia-induced renal injury. **(A)** H and E pathological staining and Masson staining of model control (SERI group), oral AST (AST-H), and AST-i. p. (AST-IP); **(B)** The level of uric acid in normal group, model group (SERI control), AST-L (oral AST 5 mg/kg/d), AST-H (oral AST 10 mg/kg/d), and AST-IP (AST i. p. 2 mg/kg/d); **(C)** the level of creatinine content in five groups; **(D)** urea nitrogen (BUN) content in five groups. Data are expressed as mean ± SD (*n* = 6), NS represented no significant differences, *p < 0.05, **p < 0.01 vs. SERI control group.

Astragaloside IV treatment was performed simultaneously in the HN model. H&E and Masson staining was applied to monitor renal histopathological changes. Histological analysis (Figure 3A) revealed that oral AST inhibited renal interstitial broadening and fibrosis. However, the pathological results of AST-i. p. Showed no therapeutic effect on renal damage, with the administration of AST avoiding exposure to the gut microbiota. There were no significant weight changes in uric acid after treatment with AST, indicating that the mechanism of the protective effect of AST did not include reducing uric acid (Figure 3B). Oral AST significantly reduced serum creatinine and BUN levels in a dose-dependent manner (Figures 3C,D). Consistent with the pathological results, serum creatinine and BUN levels showed no significant changes after AST-i. p. Treatment.

### Astragaloside i.v. improves the pharmacokinetics of febuxostat in HN rats

**FIGURE 4:**
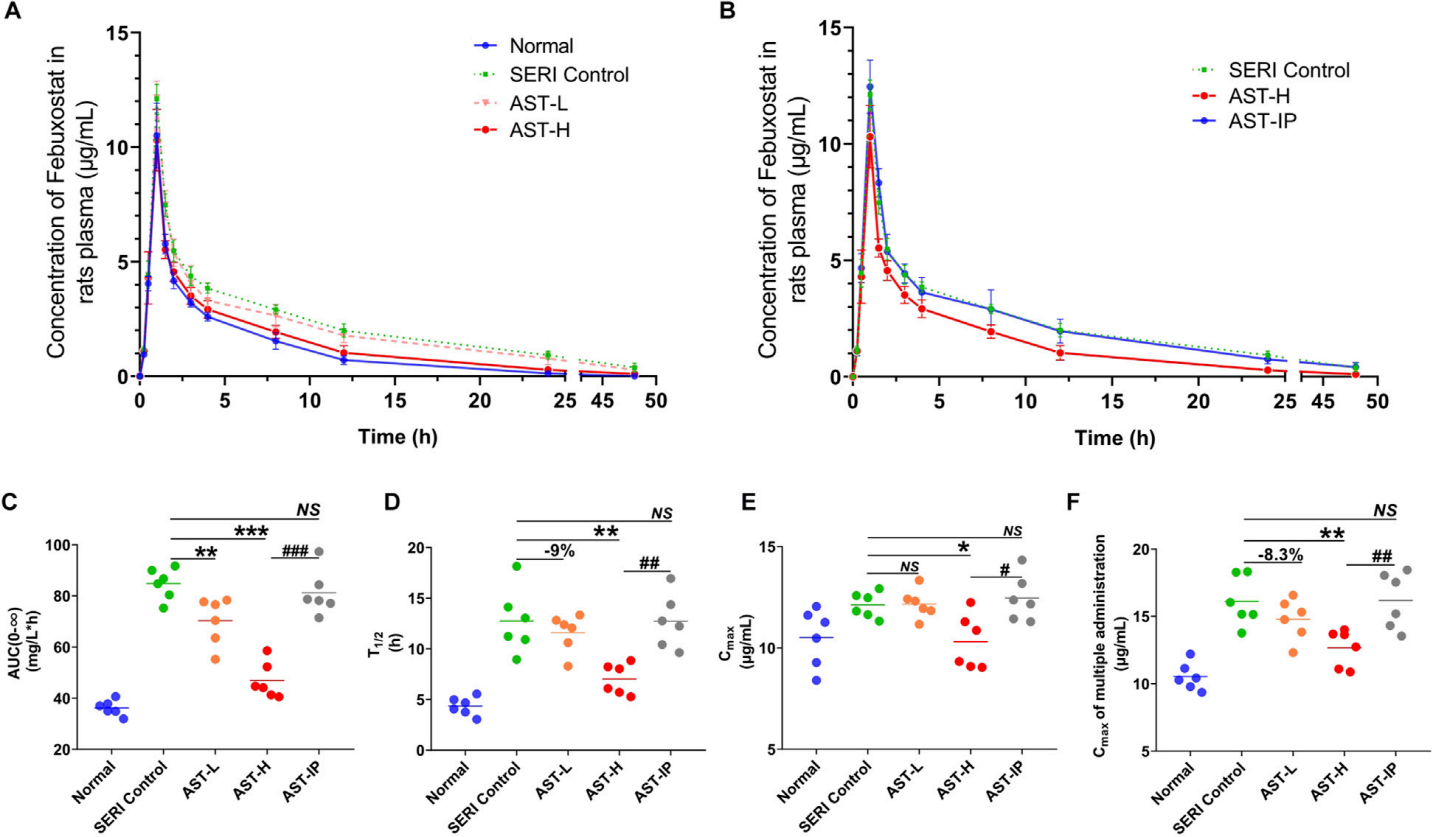
AST improves the pharmacokinetics of febuxostat in HN rats. **(A)** Pharmacokinetics of febuxostat in normal group, HN model group (SERI control), AST-L and AST-H group; **(B)** Pharmacokinetics of febuxostat in HN model group (SERI control), AST-H group (oral AST), AST-IP group (AST i. p.); **(C)** the value of AUC(0-∞) of normal group, HN model group (SERI control), AST-L group, AST-H group and AST-IP group; **(D)** the value of T_1/2_ of five groups; **(E)** the value of Cmax of five groups; **(F)** the value of C_max_ of five groups after multiple administration. NS represented no significant differences, *p < 0.05, **p < 0.01, ***p < 0.001 vs. SERI control group; ^#^ *p* < 0.05, ^##^ *p* < 0.01, ^###^ *p* < 0.001 vs. AST-H.

The pharmacokinetics of febuxostat after AST treatment were evaluated in HN rats. Febuxostat (10 mg/kg, oral) was administered to AST-L, AST-H, and AST-IP rats. The drug concentration-time curves for normal, SERI, AST-L, AST-H, and AST-IP rats are shown in Figures 4A,B. The oral AST corrected the metabolic abnormalities of febuxostat in a dose-dependent manner. However, intraperitoneal injection of AST, as the administration form avoiding exposure to the gut microbiota, was not effective in improving the pharmacokinetics of febuxostat. The pharmacokinetic parameters are shown in the supplementary material. The AUC, T_1/2_, and C_max_ (Figures 4C–E) of HN rats decreased significantly, in a dose-dependent manner, after the oral administration of AST, whereas AST (i.p.) did not reduce the values of AUC, T_1/2_, and C_max_. After the study of febuxostat pharmacokinetic, the rats were treated with febuxostat (10 mg/kg/day, oral) for continual 7 days. The C_max_ of last administration decreased significantly with the orally giving of AST in a dose-dependent manner (Figure 4F), while AST (i.p.) did not reduce the values of the C_max_.

### The T_1/2_ of febuxostat was positively correlated with serum creatinine, BUN, and ammonia

**FIGURE 5:**
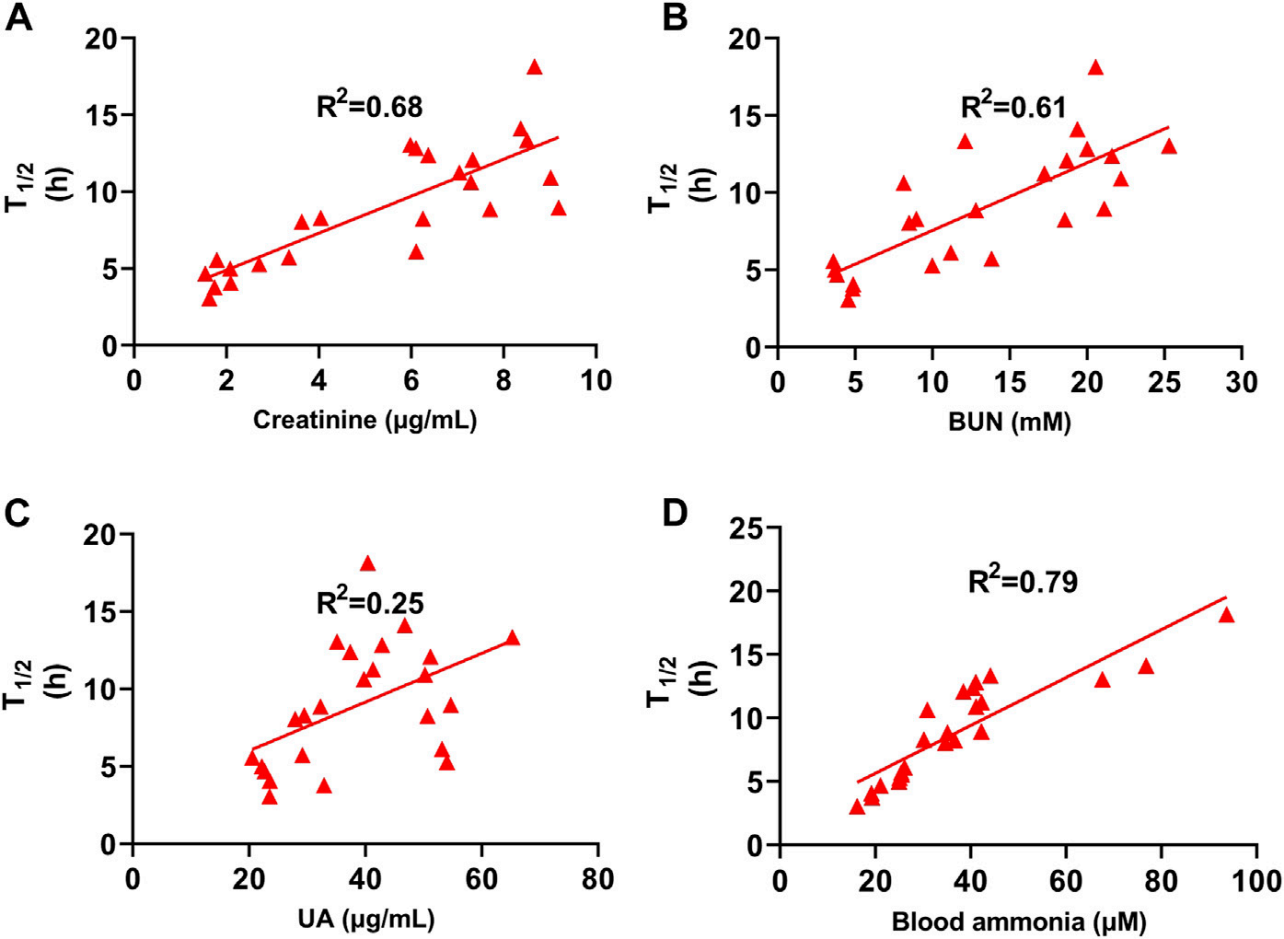
T_1/2_ of Febuxostat Positively Correlated with Serum Creatinine, BUN, and ammonia. **(A)** Correlation between T_1/2_ and creatinine of normal group, HN model group (SERI control), AST-L and AST-H group (*n* = 24); **(B)** Correlation between T_1/2_ and BUN of normal group, HN model group (SERI control), AST-L and AST-H group (*n* = 24); **(C)** Correlation between T_1/2_ and uric acid of normal group, HN model group (SERI control), AST-L and AST-H group (*n* = 24); **(D)** Correlation between T_1/2_ and blood ammonia of normal group, HN model group (SERI control), AST-L and AST-H group (*n* = 24).

We determined the relationship between biochemical indices and T_1/2_ to explore how AST improves febuxostat pharmacokinetics. The related coefficient is expressed as *R* ^2^, a general parameter in the least-squares method. The correlation between urinary acid and T_1/2_ was poor (*R* ^2^ = 0.25) (Figure 5C). The T_1/2_ of febuxostat was positively correlated with serum creatinine, BUN, and ammonia (Figures 5A,B,D), with the related coefficient *R* ^2^ values of 0.68, 0.61, and 0.79, respectively. These results imply that blood ammonia, with the strongest correlation with T_1/2_, might be the key mediator of AST febuxostat metabolism regulation.

### AST regulated urea metabolism in faeces

**FIGURE 6:**
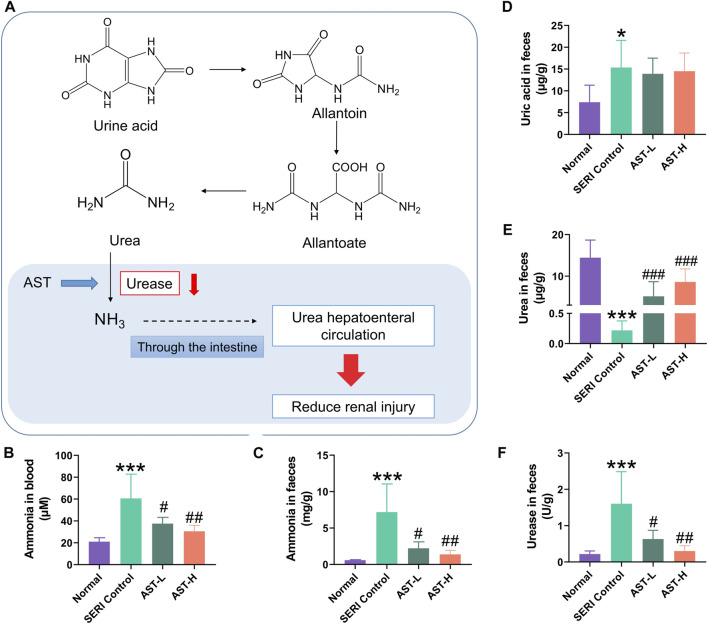
AST regulated urea metabolism in faeces. **(A)** Uric acid metabolic pathway in feaces; **(B)** the value of blood ammonia of normal group, SERI group, AST-L group, and AST-H group; **(C)** the value of fecal ammonia of normal group, SERI group, AST-L group, and AST-H group; **(D)** the value of uric acid of normal group, SERI group, AST-L group, and AST-H group; **(E)** the value of urea in faces of normal group, SERI group, AST-L group, and AST-H group; **(F)** the activity of urease in faces of normal group, SERI group, AST-L group, and AST-H group. *p < 0.05, ***p < 0.001 compared with normal group; ^#^ *p* < 0.05, ^##^ *p* < 0.01, ^###^ *p* < 0.001 compared with SERI group.

Recent studies revealed that changes in the composition and function of gut microbiota are closely related to the progression of chronic kidney disease due to abnormal urea metabolism mediated by the gut microbiota. The metabolic pathway of uric acid in faeces is uric acid → allantoin → allantoate → urea → ammonia (Figure 6A). The generated free ammonia can be absorbed back through the intestine and enter systemic circulation (a process called urea hepatoenteral circulation). Therefore, we evaluated the ammonia levels in the blood and faeces (Figures 6B,C). Ammonia in HN rats significantly increased in both blood and faeces compared with normal rats, and AST treatment significantly inhibited the increase in ammonia. Urine acid, urea, and urease were detected in faeces (Figures 6D–F). The results of validating the method of uric acid and urea quantification by LC-MS/MS are shown in Supplementary Tables S3 and 4. Urine acid in the faeces of HN rats was significantly higher than that in the faces of normal rats. However, it remained stable after AST treatment, indicating that AST could regulate blood ammonia rather than uric acid levels. Because of the abnormal increase in urease activity, the level of urea in HN rat faeces was significantly lower than that in the normal group. AST could reverse the imbalance in urea metabolism in HN rat faeces, most likely caused by the regulation of urease activity.

### Microbial diversity analysis

**FIGURE 7:**
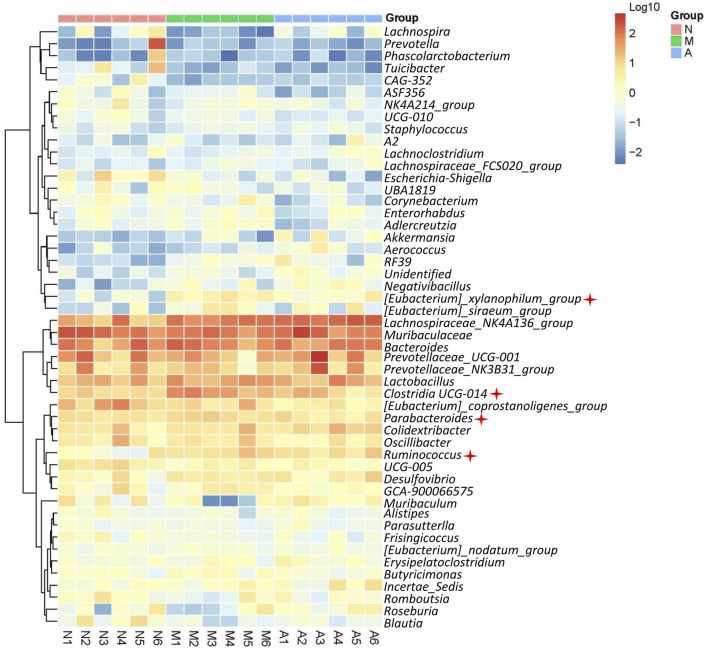
Microbial diversity analysis. Heat map showing top 50 genus of normal rats (N), model rats (SERI group, M), and AST-H group (A).

The gut microbiota composition of 18 rats was analyzed by 16 S rRNA gene sequencing. Barcoded pyrosequencing of the V3 and V4 regions of the 16 S rRNA gene showed that the gut microbiota of HN rats was dysregulated. AST could regulate the gut microbiota composition. The genera differences in the three groups are shown in the supplementary materials (Supplementary Figure S1A, B). The proportions of the main genera of each rat are shown in Supplementary Figure S1C. A heat map shows the top 50 bacterial genera that exhibited the most substantial abundance changes after exposure to AST (Figure 7). Of the 50 genera, the abundance of 13 genera (Lachnospiraceae*, Parabacteroides, Ruminococcus, Desulfovibrio, Clostridia_UCG-014, Eubacterium, Negativibacillus, Ruminococcus, Monoglobus, Gastranaerophilales, Acetatifactor, Butyricimonas*, and *Adlercreutzia*) increased and the abundance of eight genera (*Faecalibacterium, Prevotella. Lachnospira, Turicibacter, Bilophila, Escherichia-Shigella,* *Blautia, Muribaculum*) decreased in HN model rats compared with their abundance in normal rats. Among these altered genera, many were related to urea metabolism. *Eubacterium, Parabacteroides, Ruminococcus*, and *Clostridia* have been reported to possess genes for urease production (Kamiya et al., 1993; Dupuy et al., 1997; Wegmann et al., 2014; Shen et al., 2016). The increase in abundance of the above four genera might lead to hyperammonemia due to the urea metabolism enhancement in the gut microbiota. Hyperammonemia may cause renal injury, one of the mechanisms of renal injury caused by hyperuricemia.

After AST treatment, the composition of the gut microbiota of HN rats improved to a certain extent. The abundance of 6 genera (*Faecalibacterium, Lachnospira, Akkermansia, Aerococcus, Muribaculum*, and *Roseburia*) increased with a simultaneous decrease in the abundance of 12 genera (*Eubacterium, Parabacteroides, Clostridia_UCG-014, Turicibacter, Enterorhabdus, Ruminococcus, Monoglobus, Gastranaerophilales, Acetatifactor, Corynebacterium, Adlercreutzia*, and *Escherichia-Shigella*). All four genera relevant to urea metabolism were decreased in the gut microbiota after AST treatment, indicating that AST could reduce urea metabolism in faeces by regulating gut microbiota.

## Discussion

Hyperuricemic nephropathy is a common complication of hyperuricemia. The main pathological basis is the deposition of uric acid crystals in the kidneys accompanied by inflammatory reactions that impair renal function (Yang et al., 2019). This study established a hyperuricemia-induced nephropathy model using a high-purine diet (including yeast powder and adenine). After pathological evaluation, we found abnormal physiological changes in the kidneys, including uric acid crystallization, renal tubule vacuolar degeneration, interstitial broadening, and fibrosis. Biochemical indices also indicated impairment of renal function.

The pathological state of animals may change the pharmacokinetic characteristics of drugs, particularly liver and kidney damage (Ma et al., 2018). A study of the *in vivo* drug disposal process noted that some possible disease complications could lead to changes in drug metabolic characteristics. Febuxostat is the first-line drug used for the treatment of hyperuricemia. However, studies on the pharmacokinetics of HN in patients and animals are scarce. In this study, we administered febuxostat orally to HN rats, and evaluated its pharmacokinetics. Surprisingly, the T_1/2_ of febuxostat was prolonged because of impaired renal clearance induced by hyperuricemic nephropathy. Consequently, significant drug accumulation after long-term administration deserves further attention. The AUC is not sufficient to reflect renal function after a single dose of febuxostat in clinical oractice. However, after increased times of administration, drug exposure differs widely between patients with various renal functions. According to drug package insert, febuxostat does not accumulate in patients with mild renal insufficiency (Ma et al., 2018). However, the clinical significance of this accumulation has not been determined. Consequently, febuxostat dose adjustments are not required in patients with renal insufficiency. A recent study reported that patients with renal dysfunction tend to accumulate drugs and experience drug-related adverse events more frequently (Fang et al., 2021). Therefore, studies of drug accumulation in patients or animals with renal insufficiency should be further validated.

Countermeasures to the above potential drug accumulation generally include dose adjustment, excretion promotion, and the assistance of renoprotective drugs. AST is a natural saponin involved in anti-oxidation, anti-inflammation, and anti-fibrosis. AST has been widely used in the treatment of kidney diseases, such as acute kidney injury and diabetes mellitus kidney damage (Zhou et al., 2020). However, AST has low oral bioavailability (<2.5%), making it difficult to determine the material basis for renoprotection in the target organ. In this study, we successfully demonstrated the protective effects of oral AST against hyperuricemia-induced nephropathy. In contrast, i. p. AST did not show a good therapeutic effect on HN rats. The gut microbiota might mediate the inconsistent efficacy caused by the route of administration. It has been reported that some natural products exert their efficacy through the gut microbiota, including albiflorin and berberine (Zhao et al., 2018; Wang et al., 2021). A recent study showed dysregulated nitrogen circulation in the gut microbiota under HN conditions, such as enhanced uric acid decomposition and urea liver-intestinal circulation (Pan et al., 2020). Therefore, modifying the composition of the gut microbiota or discovering drugs targeting the characteristic metabolic enzymes of the gut microbiota, such as urease, could be a useful way to treat or prevent HN.

Metabolites in the uric acid pathway in faeces include uric acid, hypoxanthine, xanthine, allantoin, and urea. Hypoxanthine and xanthine are the main precursors of uric acid, and allantoin and urea are uric acid catabolic products (Pan et al., 2020). Uric acid is decomposed into urea, which is thoroughly decomposed by urease in the gut microbiota (Figure 6A). In this study, uric acid and urea levels were found to differ significantly in the faeces of HN rats. After AST treatment, urea tended to normalize, while uric acid remained unchanged, indicating that AST might protect renal function by affecting urea metabolism in faeces instead of uric acid. We determined urea’s catabolic enzymes and metabolic end-products to prove this hypothesis. The results indicated that AST could resist the decrease in urease activity in the gut microbiota caused by HN, and the level of urease was closely related to the reconstruction of gut microbiota under diseased conditions. In addition, the enhanced urease activity was directly related to the decrease in urea levels in the gut microbiota, thereby promoting urea liver-intestinal circulation (Lau and Vaziri, 2017). This leads to elevated plasma levels of ammonia, which in turn aggravates renal function damage (Varga et al., 2018). As a result, AST reduced the abnormal increase in ammonia in the blood and faeces of HN rats. Hyperammonemia has been reported to cause tubular dilation and tubulointerstitial nephritis in mice, leading to stage 3 acute kidney injury in patients (Warrillow et al., 2020). Therefore, AST could prevent hyperuricemia-induced renal injury by reducing urease activity in the gut microbiota.

The microbial diversity analysis results showed an obvious disorder in the gut microbiota of HN rats, and orally administered AST could resist this abnormal change. Of the 14 increased genera in HN rats, *Eubacterium, Parabacteroides, Ruminococcus*, and *Clostridia* were reported to possess the gene governing urease production (Kamiya et al., 1993; Dupuy et al., 1997; Wegmann et al., 2014; Shen et al., 2016). The abundant increase of the above four genera (marked in Figure 7) might lead to hyperammonemia due to the urea metabolism enhancement in the gut microbiota. Moreover, the abundance of the above four genera decreased after AST treatment, which might be the key mechanism of renal protection by AST in the gut microbiota. Furthermore, *Eubacterium nodatum* was reported to be a biomarker of chronic kidney disease. AST reduced the abnormally elevated abundance of *Eubacterium* in rats’ gut microbiota (Chen et al., 2022). Encrusted pyelitis and cystitis are peculiar disorders characterized by calcification of the vesical, pyelic, and/or ureteral walls due to the presence of *Corynebacterium urealyticum* (Sakhi et al., 2021). These calcifications are usually composed of struvite (ammonium magnesium phosphate) and calcium carbonate‒apatite crystals, caused by urea-splitting bacteria urinary infections (Flannigan et al., 2014). AST reduced the abundance of *Corynebacterium* in the gut microbiota of HN rats, exerting an anti-encrusted pyelitis effect. In addition, *Faecalibacterium* and *Roseburia* were reported to be short-chain fatty acid-generating bacteria, which increased significantly after AST treatment. Supplementation with short-chain fatty acids (SCFA) can significantly improve renal function (Marzocco et al., 2018; Zheng et al., 2020). In end-stage renal disease (ESRD), gut-derived uremic toxins play a crucial role in systemic inflammation and oxidative stress, promoting excess morbidity and mortality. Biochemical derangement is partly a consequence of the insufficient generation of SCFA due to dysbiosis of the gut and insufficient consumption of fermentable complex carbohydrates. Supplementation of ESRD patients with sodium propionate could reduce pro-inflammatory parameters and oxidative stress, improve insulin resistance and iron metabolism, and improve renal function (Marzocco et al., 2018). AST could increase the abundance of SCFA-producing bacteria in the gut microbiota, which might be another effect in relieving renal disease. In summary, the protective effect of AST on nephropathy induced by hyperuricemia results from a comprehensive regulation of the gut microbiota, including regulation of urea metabolism, anti-calcification, and short-chain fatty acid generation.

## Conclusion

Hyperuricemia nephropathy is a common complication of hyperuricemia that causes renal dysfunction in patients. In this study, HN rats were established using a high-purine diet. The pharmacokinetics of oral febuxostat were abnormal in HN rats, and drug accumulation occurred after multiple administrations of febuxostat. AST can correct abnormal pharmacokinetics of febuxostat *via* renal protection. Microbial diversity analysis revealed that the number of bacterial strains was diminished or increased in the model group. In addition, there was dysregulated nitrogen circulation in the gut microbiota under diseased conditions, such as enhanced uric acid decomposition pathway and urea liver-intestinal circulation. AST can regulate the urea metabolism of gut microbiota by targeting urease due to the gut microbiota’s modified structure. In summary, AST improved febuxostat pharmacokinetics in HN rats by comprehensive regulation of the gut microbiota, including regulation of urea metabolism, anti-calcification, and generation of short-chain fatty acids. This work has provided data for the rational application of febuxostat and partly explains the pathogenesis of HN based on the gut microbiota.

## Data Availability

The datasets presented in this study can be found in online repositories. The names of the repository/repositories and accession number(s) can be found below: NCBI BioProject (https://www.ncbi.nlm.nih.gov/bioproject/), PRJNA883534.
