# Supplementary material for: Astragaloside IV improves the pharmacokinetics of febuxostat in rats with hyperuricemic nephropathy by regulating urea metabolism in gut microbiota
Source: Front Pharmacol. 2022 Dec 20;13:1031509. doi: 10.3389/fphar.2022.1031509 (PMC9807765; doi:10.3389/fphar.2022.1031509)
Supplement: Supplementary file 1 [file Table1.DOCX]

Supplementary Material

Table legends

Table S1 Accuracy and precision of febuxostat LC-MS/MS analysis

Table S2 Extraction recovery and matrix effect of febuxostat LC-MS/MS analysis

Table S3 Accuracy and precision of uric acid and urea LC-MS/MS analysis

Table S4 Extraction recovery and matrix effect of uric acid and urea LC-MS/MS analysis

Table S5 Pharmacokinetic parameters of febuxostat

Table S6 Pharmacokinetic parameters of single and multiple administration

Table S1 Accuracy and precision of febuxostat LC-MS/MS analysis

| Nominal Concentration  (μg/mL) | Accuracy | | | | Intra-day | | Inter-day | |
| --- | --- | --- | --- | --- | --- | --- | --- | --- |
|  | Mean | SD | RE (%) | RSD (%) | Mean | RSD (%) | Mean | RSD (%) |
| LLOQ (0.05) | 0.05 | 0.01 | 96.11 | 13.23 |  |  |  |  |
| LQC (0.1) | 0.10 | 0.01 | 98.28 | 13.34 | 0.10 | 5.88 | 0.10 | 8.07 |
| MQC (5) | 5.09 | 0.46 | 101.84 | 10.10 | 5.17 | 9.57 | 5.04 | 7.36 |
| HQC (16) | 15.89 | 0.71 | 99.37 | 4.67 | 15.60 | 2.93 | 15.93 | 3.52 |

Table S2 Extraction recovery and matrix effect of febuxostat LC-MS/MS analysis

| Nominal Concentration  (μg/mL) | Extraction Recovery | | Matrix Effect | |
| --- | --- | --- | --- | --- |
|  | (%) | RSD (%) | (%) | RSD (%) |
| LQC (0.1) | 109.25 | 6.02 | 105.54 | 6.57 |
| MQC (5) | 101.83 | 5.40 | 102.87 | 5.99 |
| HQC (16) | 99.61 | 2.71 | 99.62 | 2.70 |

Table S3 Accuracy and precision of uric acid and urea LC-MS/MS analysis

| Analytes | Nominal Concentration | Accuracy | | | | Intra-day | | Inter-day | |
| --- | --- | --- | --- | --- | --- | --- | --- | --- | --- |
|  | (μg/mL) | Mean | SD | RE (%) | RSD (%) | Mean | RSD (%) | Mean | RSD (%) |
| uric acid | LLOQ (0.1) | 0.10 | 0.01 | 96.7 | 11.80 |  |  |  |  |
|  | LQC (0.2) | 0.19 | 0.01 | 97.0 | 5.85 | 0.19 | 6.52 | 0.20 | 5.81 |
|  | MQC (20) | 20.21 | 0.93 | 101.0 | 4.60 | 20.70 | 3.76 | 20.61 | 3.91 |
|  | HQC (80) | 81.11 | 3.19 | 101.4 | 3.93 | 81.76 | 3.47 | 80.89 | 3.64 |
| urea | LLOQ (0.1) | 0.11 | 0.01 | 106.3 | 8.59 |  |  |  |  |
|  | LQC (0.2) | 0.19 | 0.02 | 97.2 | 8.78 | 0.20 | 8.93 | 0.19 | 7.50 |
|  | MQC (20) | 20.42 | 1.08 | 102.1 | 5.30 | 19.80 | 8.99 | 19.68 | 6.86 |
|  | HQC (80) | 81.23 | 3.03 | 101.5 | 3.73 | 79.92 | 4.46 | 80.78 | 3.57 |

Table S4 Extraction recovery and matrix effect of uric acid and urea LC-MS/MS analysis

| Analytes | Nominal Concentration | Extraction Recovery | | Matrix Effect | |
| --- | --- | --- | --- | --- | --- |
|  | (μg/mL) | (%) | RSD(%) | (%) | RSD (%) |
| uric acid | LQC (0.2) | 109.3 | 6.02 | 97.23 | 6.21 |
|  | MQC (20) | 101.8 | 5.40 | 98.65 | 4.36 |
|  | HQC (80) | 99.6 | 2.71 | 98.91 | 4.19 |
| urea | LQC (0.2) | 97.1 | 10.72 | 94.39 | 4.38 |
|  | MQC (20) | 97.2 | 7.03 | 95.34 | 4.75 |
|  | HQC (80) | 99.5 | 4.33 | 98.41 | 3.53 |

Table S5 Pharmacokinetic parameters of febuxostat

|  |  | Normal | | SLRI | | SERI | | AST-L | | AST-H | | AST-IP | |
| --- | --- | --- | --- | --- | --- | --- | --- | --- | --- | --- | --- | --- | --- |
|  |  | Mean | SD | Mean | SD | Mean | SD | Mean | SD | Mean | SD | Mean | SD |
| AUC(0-t) | mg/L*h | 35.70 | 2.60 | 43.58 | 4.47 | 78.80 | 4.92 | 66.35 | 8.22 | 46.39 | 6.61 | 75.71 | 8.74 |
| AUC(0-∞) | mg/L*h | 36.22 | 2.97 | 44.33 | 4.08 | 84.80 | 6.15 | 70.28 | 9.28 | 46.94 | 7.08 | 81.15 | 8.92 |
| MRT(0-t) | h | 5.48 | 0.63 | 6.40 | 1.33 | 12.36 | 1.46 | 11.26 | 1.36 | 8.52 | 1.19 | 12.00 | 1.55 |
| MRT(0-∞) | h | 5.92 | 0.74 | 6.79 | 1.34 | 17.09 | 3.92 | 14.92 | 2.84 | 9.71 | 1.76 | 19.07 | 6.72 |
| t1/2z | h | 4.35 | 0.90 | 4.88 | 1.11 | 12.74 | 3.20 | 11.59 | 1.86 | 7.04 | 1.51 | 12.73 | 2.66 |
| Tmax | h | 1.00 | 0.00 | 1.00 | 0.00 | 1.00 | 0.00 | 1.00 | 0.00 | 1.00 | 0.00 | 1.00 | 0.00 |
| Cmax | mg/L | 10.51 | 1.42 | 11.22 | 1.39 | 12.12 | 0.62 | 12.16 | 0.72 | 10.31 | 1.35 | 12.46 | 1.14 |

Table S6 Pharmacokinetic parameters of single and multiple administration

|  |  | Single administration | | Multiple administration | |
| --- | --- | --- | --- | --- | --- |
|  |  | Mean | SD | Mean | SD |
| AUC(0-t) | mg/L*h | 78.80 | 4.92 | 106.01^**^ | 15.95 |
| AUC(0-∞) | mg/L*h | 84.80 | 6.15 | 123.92 | 35.64 |
| MRT(0-t) | h | 12.36 | 1.46 | 14.16 | 1.42 |
| MRT(0-∞) | h | 17.09 | 3.92 | 23.88 | 7.51 |
| t1/2z | h | 12.74 | 3.20 | 16.02 | 7.79 |
| Tmax | h | 1.00 | 0.00 | 1 | 0 |
| Cmax | mg/L | 12.12 | 0.62 | 13.84^**^ | 0.90 |

^**^, *p*<0.01

Figure legends

Figure S1 The genera differences in three groups of microbial diversity analysis. (A) PCA results based on OUTs demonstrate the differences in three groups in terms of gut microbiota composition, A (AST-H group), M (SERI group, model group), N (normal group); (B) anosim analysis of gut microbiota composition differences in three groups, A (AST-H group), M (SERI group, model group), N (normal group), R>0 indicates that the difference between groups is greater than that within groups; p<0.05 indicates that there were significant differences within groups; (C) the relative abundance of TOP 8 phylum of each samples, A (AST-H group), M (SERI group, model group), N (normal group).


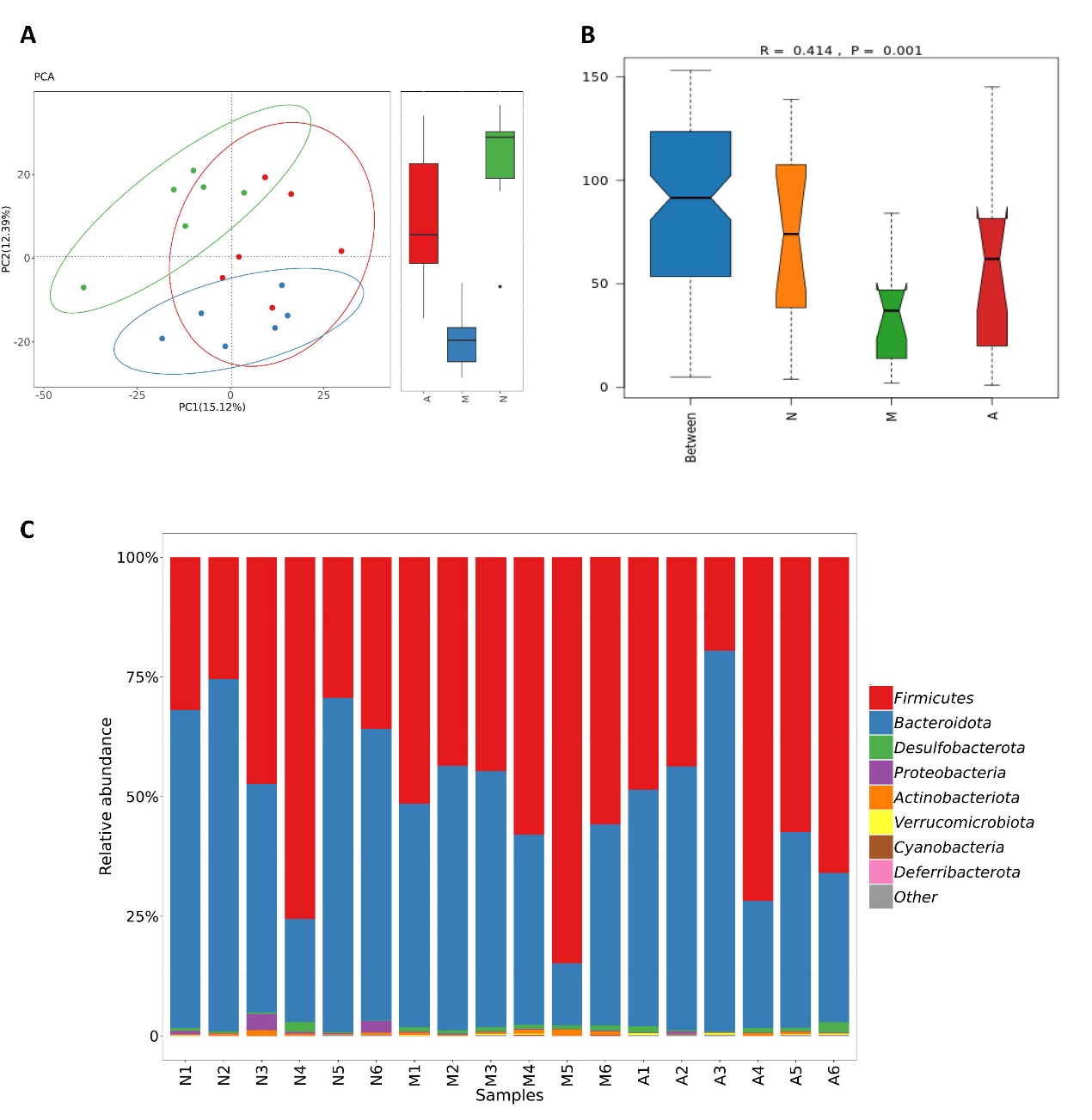


Figure S1 The genera differences in three groups of microbial diversity analysis
