# Supplementary material for: Astragaloside IV improves the pharmacokinetics of febuxostat in rats with hyperuricemic nephropathy by regulating urea metabolism in gut microbiota
Source: Front Pharmacol. 2022 Dec 20;13:1031509. doi: 10.3389/fphar.2022.1031509 (PMC9807765; doi:10.3389/fphar.2022.1031509)
Supplement: Supplementary file 2 [file DataSheet2.DOCX]

All the raw data of this study can be obtained from the follow link: https://www.jianguoyun.com/p/DUtrHAYQxc3yChj34NUEIAA
